# Dynamic interaction of local and transhemispheric networks is necessary for progressive intensification of hippocampal seizures

**DOI:** 10.1038/s41598-018-23659-x

**Published:** 2018-04-04

**Authors:** Fredrik Berglind, My Andersson, Merab Kokaia

**Affiliations:** Epilepsy Centre, Department of Clinical Sciences, Lund University Hospital, Lund, Sweden

## Abstract

The detailed mechanisms of progressive intensification of seizures often occurring in epilepsy are not well understood. Animal models of kindling, with progressive intensification of stimulation-induced seizures, have been previously used to investigate alterations in neuronal networks, but has been obscured by limited recording capabilities during electrical stimulations. Remote networks in kindling have been studied by physical deletions of the connected structures or pathways, inevitably leading to structural reorganisations and related adverse effects. We used optogenetics to circumvent the above-mentioned problems inherent to electrical kindling, and chemogenetics to temporarily inhibit rather than ablate the remote interconnected networks. Progressively intensifying afterdischarges (ADs) were induced by repetitive photoactivation of principal neurons in the hippocampus of anaesthetized transgenic mice expressing ChR2. This allowed, during the stimulation, to reveal dynamic increases in local field potentials (LFPs), which coincided with the start of AD intensification. Furthermore, chemogenetic functional inhibition of contralateral hippocampal neurons via hM4D(Gi) receptors abrogated AD progression. These findings demonstrate that, during repeated activation, local circuits undergo acute plastic changes with appearance of additional network discharges (LFPs), leading to transhemispheric recruitment of contralateral dentate gyrus, which seems to be necessary for progressive intensification of ADs.

## Introduction

An accumulating body of evidence supports the idea that interaction between neuronal networks and systems is crucial for normal brain function. These network interactions are occurring at various frequency oscillations, possibly specifying the temporal windows of increased and reduced excitability, supporting information processing in the brain^1^. Understanding mechanisms behind these oscillations and their alterations is also important for elucidating brain dysfunction in neurological disorders. In epilepsy, pathologic hyper-synchronized oscillations of the networks are manifested as seizures. About 1% of the world population suffers from epilepsy, reducing their quality of life^2^. Temporal lobe epilepsy (TLE) is the most common form in adults, where seizures originate in temporal cortical structures of the brain, particularly the hippocampus. TLE is often refractory to currently available anti-epileptic drugs, comprising more than 30% of cases^3,4^. In order to better understand mechanisms of ictogenesis and epileptogenesis in the temporal lobe, it is necessary to delineate the components of hypersynchronized network oscillations in the hippocampus, and determine how seizures intensify, progress and become recurrent in chronic TLE.

A major question still unresolved is how local neuronal circuits are activated to trigger plastic reorganization of the network, rendering it more prone to generate progressive recurrent seizures. This question has historically been tackled using electrical stimulation models, starting with *in vivo* kindling^5^ and later rapid kindling models^6,7^, as well as their *in vitro* variants^8^. However, lack of cell-population specificity, as well as indiscriminative activation of both local neurons and passing-through fibers, remained major limitations of electrical stimulation in pinpointing the specific neuronal networks responsible for focal epileptogenic plasticity. Moreover, the same electrodes were used to electrically stimulate local areas, switching to recording mode after stimulation, which made it impossible to observe electrographic alterations in local networks during stimulation. Optogenetic tools circumvent these limitations^9,10^ as well as enable nearly artifact-free recording during the stimulation period^11^. Therefore, this technology could help in delineating, during stimulations, the alterations in local circuits necessary to trigger plasticity leading to progressive afterdischarges (ADs), and potentially inform us on how to interfere with this process in more specific ways. Encouraging such an approach, optogenetic stimulation has been shown to induce seizure activity *in vivo*^12,13^.

The main objectives of the present study were to investigate whether minimal optogenetic stimulation (3-30 times lower power than previous studies, cited above) in transgenic mice would reliably elicit progressive intensification of ADs, and to delineate the changes induced in local and associated remote neuronal networks contributing to plasticity and increased excitability in the hippocampus.

Combining optogenetic stimulation of the hippocampus and chemogenetic inhibition of contralateral hippocampus (via the hM4D (modified human muscarinic M_4_) receptor, also known as an inhibitory “DREADD”)^14–16^, we demonstrate dynamic changes in local and transhemispheric networks, and necessity of contralateral dentate activation for progressive increase in excitability and intensification of ADs.

## Results

### Optogenetic stimulation in the hippocampus of Thy1-ChR2 mice induces progressively intensifying ADs

**Figure 1:**
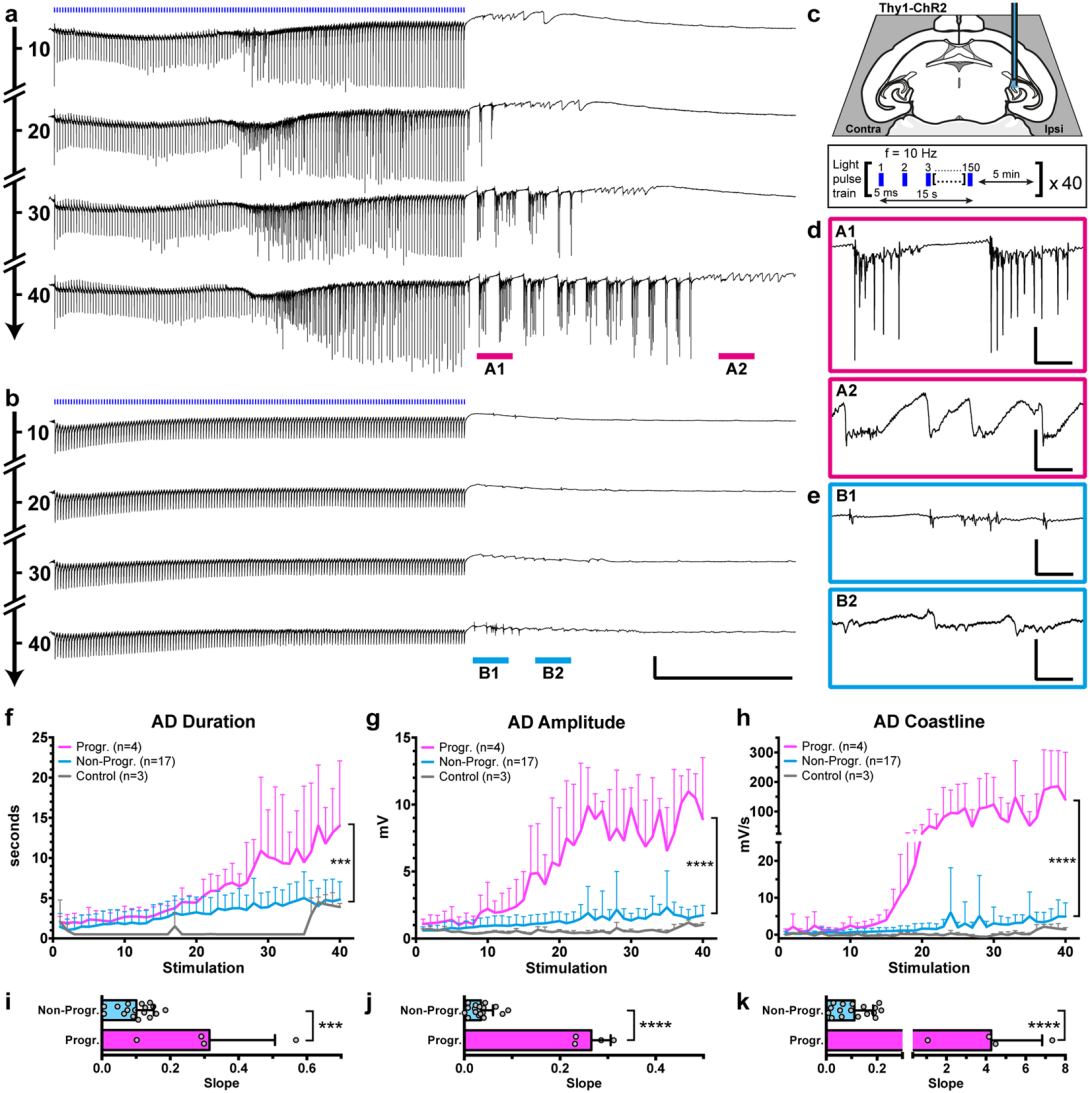
Two main patterns of progressive or non-progressive afterdischarges (ADs) induced by repetitive pulse-train photostimulation in the Thy1-ChR2 mouse *in vivo*. (**a,b**) Isoflurane anaesthetized mice were subjected to 15 s blue light (463 nm) pulse trains. Representative *in vivo* local field recordings from the hippocampus of two mice are shown, number of stimulations indicated on the timeline arrow (left). (**c**) Top: illustration of unilateral stimulation & recording area in horizontal section, with optrode targeting CA3 at DV-2.75. Bottom: pulse train parameters. (**d**,**e**) Magnified views of ADs marked in (**a** & **b**) respectively. (**f–h**) Three AD parameters in the first 5 seconds after photostimulation were plotted, grouped by progressive (Progr, n = 4) or non-progressive (Non-Progr, n = 17) AD development. Comparing the mean of the last 5 trains, the two subsets were different for all three AD metrics (unpaired t-test). A control group (n = 3, grey) did not display any afterdischarge activity when subjected to orange (593 nm) light (see Methods), while blue light administered for the last 5 stimulations produced only limited non-progressive ADs. (**i–k**) Within-subject analysis using linear regression slopes of the data in (**f**–**h)** was performed. Increased slope was seen in the Progr AD group (unpaired t-tests). Note that two-segment axes with 10 times scale of bottom segment is used for coastline in (**h** & **k**) to be able to discern Non-Progr data points. Scale bars: (**a,b**) 3 mV, 5 s; (**d,e**) A1 & B1 3 mV, 200 ms; A2 & B2 1 mV, 200 ms. Error bars: ±SD. ***P < 0.001, ****P < 0.0001, see Results for exact *P*-values.

Optogenetic activation of hippocampal principal neurons was performed in anaesthetized Thy1-ChR2 transgenic mice. ChR2 expression in these mice is mainly found in pyramidal/excitatory projection neurons^17,18^. Blue light pulse-trains induced local field potentials (LFPs) and development of associated post-train ADs, both varying considerably in magnitude between animals. Two major patterns of light-induced responses emerged (examples in Fig. 1a,b): (i) with several, regularly occurring intra-train post-light LFPs (in-between single light pulses) of variable amplitude (see next section), followed by post-train ADs that progressively and dramatically increased in both amplitude (≥5 mV) and duration (≥5 s) over the course of 40 light stimulation sessions; (ii), a majority of cases, with few or no intra-train post-light LFPs, followed by low amplitude, non-progressive ADs of shorter duration. The pulse-trains were targeted to the CA3 subfield of ventral (temporal) hippocampus (Fig. 1c). The illumination parameters were based on preliminary pilot experiments, testing various stimulation frequencies and pulse widths, and are consistent with previously published data^13^. Low light power was used to minimize the volume of the light-activated tissue: the approximated volume of activated tissue at the light power used is less than 0.2 mm^3^ (see Methods). The ADs in both subsets of animals (i and ii) were characterized by an initial phase of burst spiking (15–30 Hz) with high amplitude (although lower overall amplitude and shorter duration in the second (ii) group), and a secondary phase of low amplitude slow waves (Fig. 1d,e). From the AD phenomenology, we could thus distinguish between progressive (Progr.) and non-progressive (Non-Progr.) ADs, based on three read-outs of AD severity: duration, amplitude and coastline (Fig. 1f–h). The Progr. group (n = 4) demonstrated clear, several-fold increase in all three AD metrics compared to the Non-Progr. group (n = 17), averaged for the last 5 trains: duration (s) 12.8 ± 6.0 vs 4.6 ± 2.3 (*P* = 0.0002), amplitude (mV) 9.7 ± 1.9 vs 1.7 ± 0.6 (*P* < 0.0001), coastline (mV/s) 152.0 ± 102.8 vs 4.1 ± 2.9 (*P* < 0.0001); t-tests, t = 4.6, 15.4 & 6.5, respectively; df = 19. Note the sharp increase of amplitude and coastline after 15–20 pulse-train stimulations in the Progr. group. A control group (grey, Fig. 1f–h) did not display any AD activity when exposed to yellow light pulse-trains, and only limited ADs similar to the Non-Progr. group when blue light was administered for the final five stimulations. To adress overall dynamics of ADs over the 40 pulse-train stimulations, within-subject modeling was used, calculating the slope of the increase for each animal, and then comparing the average of slopes between the groups (Fig. 1i–k). The analysis demonstrated that ADs in the Progr. group had steeper slopes (indicating progressive intensification) compared to the Non-Progr. group: for duration 0.31 ± 0.19 vs 0.10 ± 0.05 (*P* = 0.0004), max amplitude: 0.27 ± 0.04 vs 0.035 ± 0.025 (*P* < 0.0001), and coastline: 4.26 ± 2.57 vs 0.11 ± 0.07 (*P* < 0.0001) (t-tests, t = 4.3, 14.8 & 7.3, respectively; df = 19). Anaesthesia depth as determined by breathing rate (breaths per minute, BPM) was similar in both Progr and Non-Progr groups: 52.8 ± 4.4 vs 52.9 ± 7.3 BPM (*P* = 0.9816, t-test).

### Differences in dynamics of evoked LFPs during optogenetic stimulation trains in Thy1-ChR2 mice

**Figure 2:**
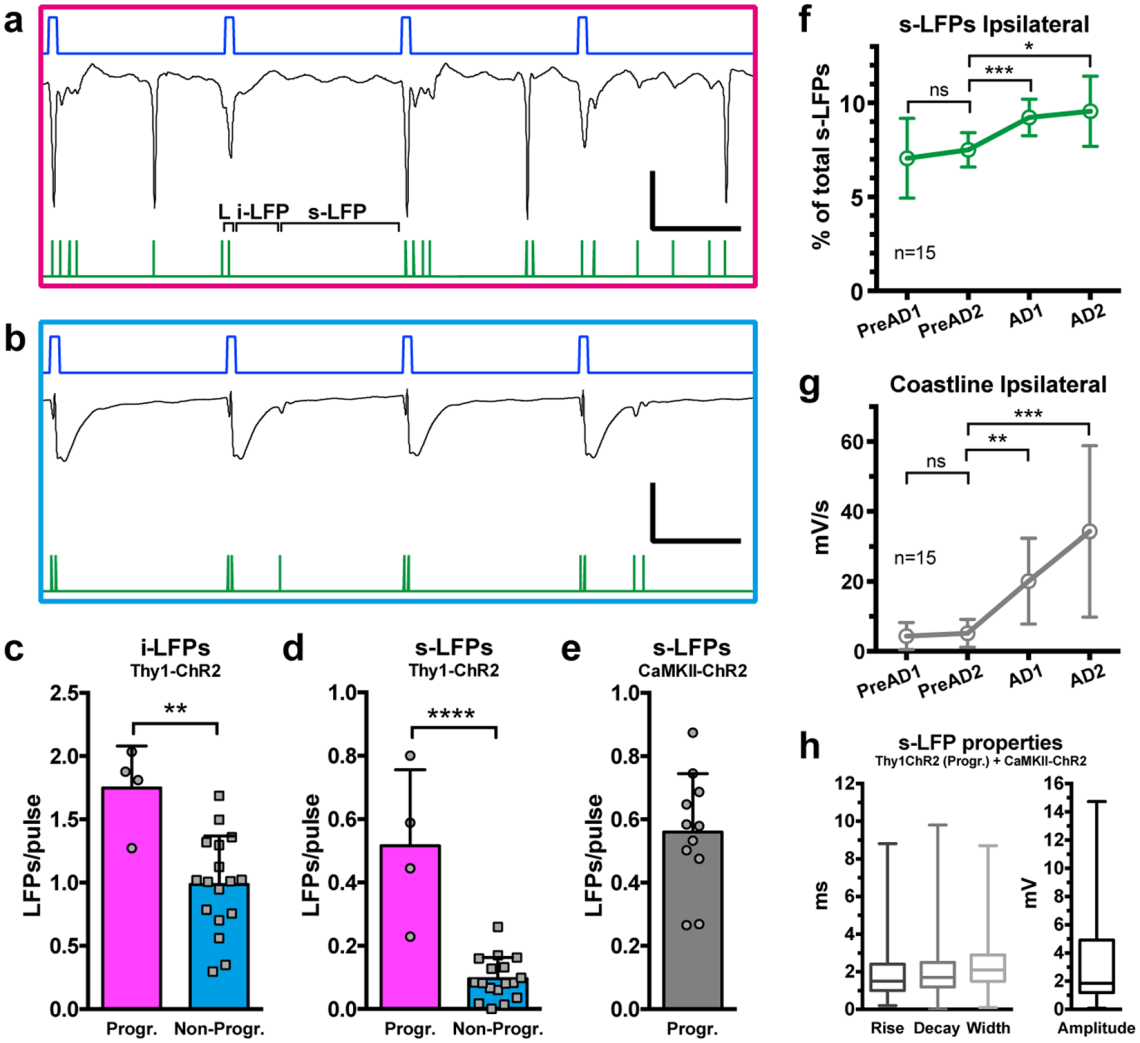
Increased post-light and spontaneous LFPs generated during the optogenetic pulse train is correlated with progressive AD occurrence. (**a,b**) Magnification of intra-train local field recordings from the 40th stimulation of two animals with progressive (**a**) and non-progressive (**b**) ADs. Blue traces: light pulses. Green traces: detected LFP events, categorized as induced (i-LFP) or spontaneous (s-LFP), while directly light-evoked LFPs, coinciding with the light pulse (L), were not included in analysis (see Results). (**c,d**) The number of i-LFPs and s-LFPs per light pulse was increased in the Progr AD group compared to the Non-Progr AD group (unpaired t-tests), especially s-LFPs. (**e**) In CaMKII-ChR2 mice with progressive light-induced ADs (n = 11, see Fig. 4), the amount of s-LFPs generated per light pulse was not different from the Thy1-ChR2 Progr group (n = 4, unpaired t-test). Thus groups were pooled for further analysis. (**f,g**) Aligning data by the stimulation producing the first progressive ipsilateral AD (see Results), a 20% increase in s-LFPs (**f**, repeated measures ANOVA with Dunnett’s post-hoc test) coincides in time with the start of progressive AD generation (**g**, Friedman test with Dunn’s post-hoc test). Binned data, three stimulations per bin; PreAD: consecutive bins preceding the first progressive AD; AD: consecutive bins from the first progressive AD. Asterisks: significance in post-hoc tests. (**h**) Boxplots of s-LFP characteristics for the pooled progressive AD group (1115 data points from 15 animals). Line: median, box: 25–75th percentile, bars: min/max. Scale bars: (**a** & **b**) 3 mV, 50 ms. Error bars: ±SD, except (**h**). *P < 0.05, **P < 0.01, ***P < 0.001, ****P < 0.0001, ns: non-significant; see Results for exact *P*-values.

We distinguished between three types of LFPs evoked within the light pulse trains. First, the high-amplitude immediate-onset LFP directly time-locked to the blue light pulse, presumably a result of synchronized activation of ChR2 channels in a large number of neurons in the vicinity of the optrode (“L”, Fig. 2a). These L-LFPs invariably appeared in all animals, and were not included in the further detailed analyses. Second, immediately following the L-LFP, a slow LFP could be observed, usually lasting for about 30 ms, during which faster post-light “induced LFPs” occurred (i-LFP, Fig. 2a). Third, “spontaneous LFPs” (s-LFP, Fig. 2a), occurring before the next light pulse. The number and magnitude of i-LFPs and s-LFPs varied dramatically between animals with progressive (Fig. 2a) and non-progressive (Fig. 2b) ADs, especially in regards to s-LFPs, and shaped the overall dynamic appearance of the LFPs during the course of pulse train stimulation. There were around 75% more i-LFPs per light pulse in the Progr. group compared to Non-Progr. group: 1.75 ± 0.33 vs 0.98 ± 0.38 (*P* = 0.0017, t-test, t = 3.7, df = 19; Fig. 2c) while the amount of s-LFPs per pulse was five-fold higher: 0.52 ± 0.24 vs 0.096 ± 0.067 (*P* < 0.0001, t = 6.6, df = 19; Fig. 2d).

### Correlation of intra-train s-LFPs and onset of progressive ADs ipsilaterally

The visual phenomenology of the LFP recordings suggested that there was an increase in the number of intra-train LFPs, especially s-LFPs, at the time of progressive AD onset (see Fig. 1a). To further quantify this observation, we also counted s-LFPs in pulse-train stimulated CaMKII-ChR2 mice displaying progressive AD development (see further in other sections below). The number of evoked ipsilateral s-LFPs per light pulse in this group (Fig. 2e) was not different from the Thy1-ChR2 Progr. group: 0.56 ± 0.18 (*P* = 0.7064, t-test), and they were therefore pooled together. We aligned 12 pulse trains in the pooled group (n = 15) to the train with the first progressive AD, and s-LFPs (normalized to the total number over the 12 trains) of every three stimulations were binned. Thus, in Fig. 2f, PreAD1 & 2 constitute the six trains leading up to the first progressive AD appearance, and AD1 & 2 correspond to the six trains from the first progressive AD. The number of s-LFPs increased by 23% & 27% compared to the bin prior to progressive AD onset; 7.5 ± 0.9 vs. 9.2 ± 1.0 & 9.6 ± 1.8 (*P* = 0.007, repeated measures ANOVA (F = 7.1, df = 14) with Dunnett’s post-hoc test: *P* = 0.0007 & 0.0179, respectively). This coincided with the marked coastline increase seen ipsilaterally at AD onset (Fig. 2g): 5.1 ± 4.0 mV/s vs 20.1 ± 12.3 & 34.3 ± 24.5 mV/s (*P* < 0.0001, Friedman test (F-stat 38.6) with Dunn’s post-hoc test: *P* = 0.0056 & 0.0001, respectively). Although the detected s-LFPs constituted a heterogeneous group, they displayed fast kinetics with a median (and 25–75% percentiles) of 1.5 (1.0–2.4) & 1.2 (1.2–2.5) ms for rise and decay times, width of 2.1 (1.5–2.9) ms and amplitude of 1.85 (1.2–4.9) mV (Fig. 2h); 1115 detected s-LFPs in total, one train from each animal.

The average lag of the first s-LFP in relation to each light pulse was 45.8 ± 9.1 ms (n = 15), but they did not occur in a stereotyped way (i.e. not time-locked to the light-pulse, see Supplementary Fig. S1). However, over the course of stimulation the s-LFPs started to appear as rhythmic bursts (usually ≤1 s, but occasionally up to 2–3 s), with a peak frequency of 76.2 ± 12.9 Hz, (Supplementary Fig. S1). In general, such rhythmic bursts of s-LFP patterns appeared around 5 seconds into the stimulation train (none were seen prior to 3 seconds).

### Fos expression in the dentate gyrus revealed different patterns of neuronal activation

**Figure 3:**
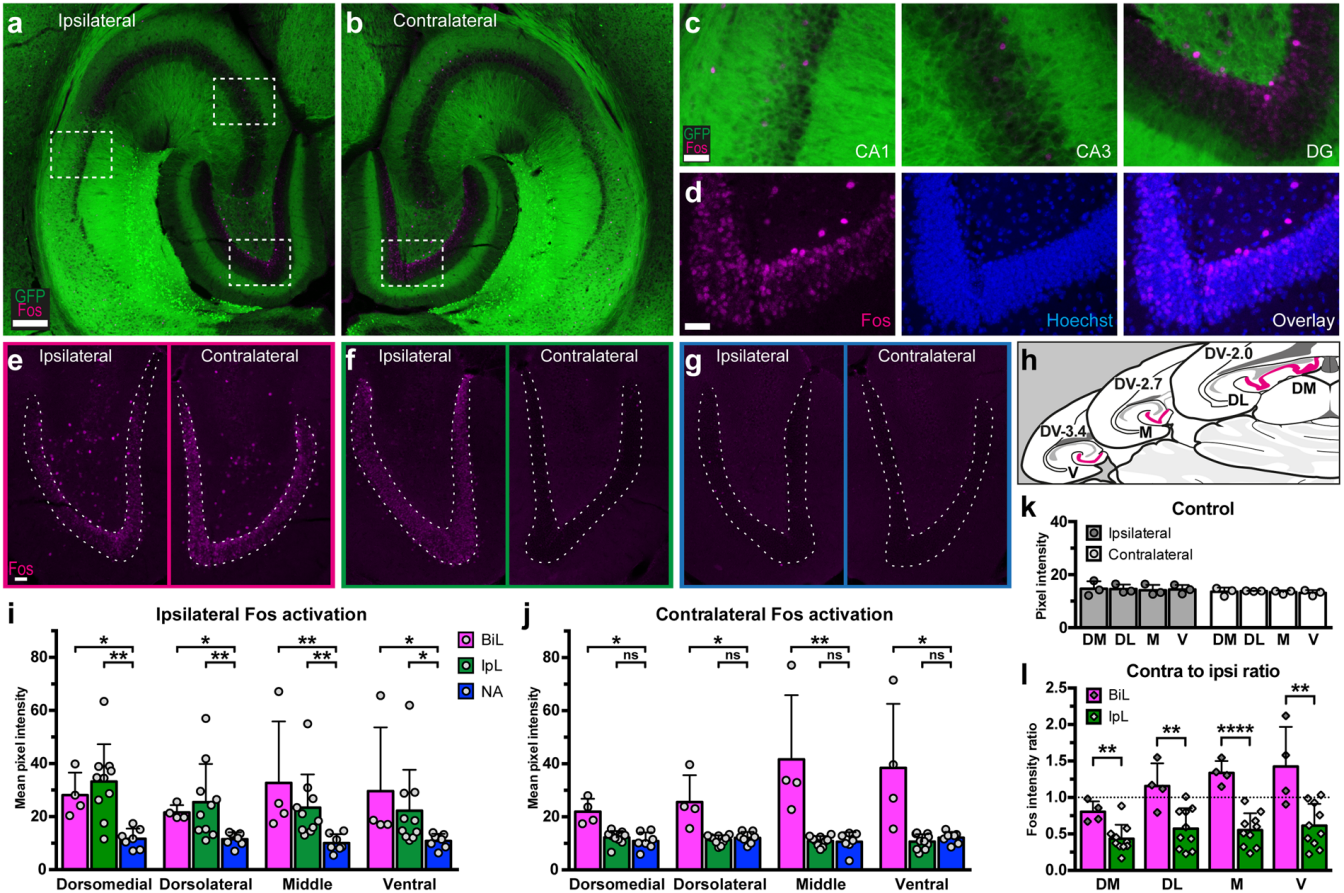
Differences in Fos immunoreactivity patterns in Thy1-ChR2 mice subjected to optogenetic stimulation in the hippocampus implies importance of contralateral DG. (**a**,**b**) Composite images of Fos immunoreactivity in horizontal sections of the hippocampus ipsilateral (**a**) and contralateral (**b**) to optogenetic stimulation from a mouse with progressive AD development. (**c**) Magnified areas from (**a**) of CA1, CA3 & DG, respectively. (**d**) Magnified area of DG from (**b**), indicating cell body localization of Fos by nuclear stain (Hoechst, blue). (**e–g**) Three general patterns of Fos immunoreactivity in the DGC (dashed outlines): (**e**) bilateral (BiL); (**f**) ipsilateral to optogenetic stimulation (IpL), (**g**) absence of Fos immunoreactivity bilaterally (non-activated, NA) (**e** & **g** are from the same animals as recordings in Fig. 1a & b, respectively). (**h**) Illustration of areas selected for quantification of Fos immunoreactivity. DGC marked in magenta. DM: dorsomedial, DL: dorsolateral, M: middle, V: ventral. (**i**) Ipsilateral and (**j**) contralateral mean Fos intensity in the BiL (n = 4), IpL (n = 10) and NA (n = 7) subsets (NA as control, Kruskal-Wallis test with Dunn’s post-test). Note that the BiL group completely matches the Progr AD group (Figs 1 & 2). (**k**) Control group (n = 3) exposed to trains of orange (593 nm) light. (**l**) Relative magnitude (ratio) of contra- to ipsilateral Fos intensity, comparing BiL and IpL (unpaired t-tests). Scale bars in a,b: 200 µm, c,d: 40 µm, e–g: 50 µm. Error bars: ±SD. *P < 0.05, **P < 0.01, ****P < 0.0001, ns: non-significant, see Table 1 and Results for exact P-values.

To identify the minimal neuronal pool activated by light illuminations and ADs, we analyzed Fos immunoreactivity in the hippocampi of all optogenetically stimulated Thy1-ChR2 animals. The activation of immediate early gene *c-fos* and its gene product Fos has been widely used as a surrogate marker for repetitive neuronal activation, including seizures, in various animal models^19–21^. In all animals, cells with Fos immunoreactivity were sparsely found dispersed in hippocampal fields of CA1 and CA3 (Fig. 3a–c), and also in the hilus of the Progr group (Fos visualized by Cy5, pseudocoloured for magenta and ChR2 by YFP autofluorescence, GFP filter, green). Notably, dense Fos expression was found almost exclusively in the dentate gyrus (Fig. 3c,d), and the focus of analysis was directed towards this area. There appeared to be a pattern of bilateral Fos expression in the DG granule cell layer (DGC) of the Progr group (Fig. 3e) while expression was limited to the ipsilateral side, or not present at all, in the Non-Progr group (Fig. 3f,g). Quantitative analysis of Fos immunoreactivity was performed on horizontal sections by measuring Cy5 pixel intensity in the DGC of four regions in the dorso-ventral (i.e. septo-temporal) axis of the hippocampi (see Fig. 3h**)**: dorsomedial (DM), dorsolateral (DL), middle (M) and ventral (V) regions. Pictures in Fig. 3a–g are from the M region. We found that a control group of animals perfused 15 minutes after receiving a single pulse train of blue light (n = 6, not shown), displayed a similar lack in overall DGC Fos immunoreactivity as a subset of Non-Progr animals (n = 7, non-activated (NA) group, Fig. 3g,i,j); Fos control vs NA: overall mean pixel intensity 12.3 ± 0.9 vs 11.0 ± 2.9 and 11.1 ± 1.0 vs 11.0 ± 2.9 for the four regions combined, ipsi- and contralateral hemispheres, respectively (*P* = 0.33 & 0.81, t-tests, t = 1.03 & 0.251; df = 11). A control group that received pulse-trains of yellow-amber light (593 nm, see Methods), which does not activate ChR2 and therefore did not produce any ADs (Fig. 3k, n = 3), also lacked Fos reactivity and was comparable to the NA group: overall ipsilateral 14.4 ± 1.7 and contralateral 13.5 ± 0.8 vs NA (*P* = 0.09 & 0.24, t-tests, t = 1.90 & 1.28, df = 8). The remaining Thy1-ChR2 mice demonstrated bilateral (BiL, n = 4) or ipsilateral only (IpL, n = 10) Fos immunoreactivity, which was significantly higher compared to the NA group in all regions ipsilaterally, while contralaterally the BiL group also had higher reactivity (Kruskal-Wallis test with Dunn’s post-hoc test, see Table 1 and Fig. 3i,j). An alternative way to differentiate the BiL and IpL groups is to compare the ratio of contralateral to ipsilateral immunoreactivity levels. The ratio of contralateral activation was higher in all regions for the BiL group vs IpL, Fig. 3l (DM, DL, M & V, respectively: *P* = 0.005, 0.005, < 0.0001 & 0.0034; t = 3.43, 3.43, 6.18 & 3.64; df = 12, unpaired t-tests). However, in both analyses above, no differences along the septo-temporal axis could be detected (not shown). It should be stressed here that the four animals of the BiL group completely match the Progr AD group defined in the first section. Additionally, we could now subdivide the Non-Progr group into IpL and NA animals. While animals in the IpL group did tend to display stronger ADs than the NA group in a few instances (contributing to the high standard deviation seen for amplitude and coastline at certain stimulations in Fig. 1g–h, blue traces), splitting them up and comparing AD metrics of all three groups did not yield any significant differences between these two groups (not shown). Taken together, these data indicate that the light pulse-trains themselves did not give rise to Fos immunoreactivity in the hippocampus, and while both progressive and non-progressive ADs could induce DGC Fos activation ipsilaterally, only progressive intensification of ADs may require concomitant activation of contralateral DG.

**Table 1:** Dentate granule cell Fos immunoreactivity levels in Thy1-ChR2 mice.

Hippocampal level	Hemisphere & K-W test	K-W stat.	Fos immunoreactivity per group (intensity, 95% CI)		
			Bilateral (n = 4)	Ipsilateral (n = 10)	No Activity (n = 7)
Dorso-medial	Ipsilateral *P* = 0.0007	11.35	26.1 (20.4–39.9) *P* = 0.0434*	34.6 (17.5–39.0) *P* = 0.0023**	11.7 (7.1–17.4)
	Contralateral *P* = 0.0023	10.04	21.1 (17.4–28.0) *P* = 0.0038**	12.8 (10.5–14.3) *P* = 0.7646	10.2 (5.9–15.1)
Dorso-lateral	Ipsilateral *P* = 0.0027	9.827	20.5 (19.7–25.6) *P* = 0.0348*	22.5 (13.2–41.8) *P* = 0.0072**	11.9 (7.0–14.2)
	Contralateral *P* = 0.0035	9.500	23.5 (15.6–39.7) *P* = 0.0263*	11.9 (8.9–12.9) *P* > 0.9999	12.4 (8.7–14.8)
Middle	Ipsilateral *P* = 0.0003	12.34	23.1 (17.4–67.1) *P* = 0.0047**	19.7 (14.5–30.9) *P* = 0.0060**	8.6 (5.4–14.7)
	Contralateral *P* = 0.0037	9.430	33.4 (22.7–77.1) *P* = 0.0237*	11.1 (8.5–12.7) *P* > 0.9999	11.0 (3.5–13.9)
Ventral	Ipsilateral *P* = 0.0034	9.573	17.8 (17.0–65.6) *P* = 0.0117*	16.6 (12.1–29.2) *P* = 0.0233*	11.4 (6.3–14.0)
	Contralateral *P* = 0.0028	9.784	33.4 (15.4–71.5) *P* = 0.0356*	10.8 (7.7–13.7) *P* = 0.9495	12.9 (8.6–15.2)

Due to data distribution, Kruskal-Wallis test was used, P-values and K-W statistic for overall tests in 2^nd^ & 3^rd^ columns. Data (averaged 8-bit pixel intensity) is presented as median with 95% CI. Dunn’s multiple comparisons test was performed with NA assigned as control, and statistically significant differences in the post-hoc tests are denoted with asterisks: *p < 0.05, **p < 0.01. See also Fig. 3i,j.

### Optogenetic hippocampal stimulation in CaMKIIa-ChR2 mice induces ADs, which spread to the contralateral side

**Figure 4:**
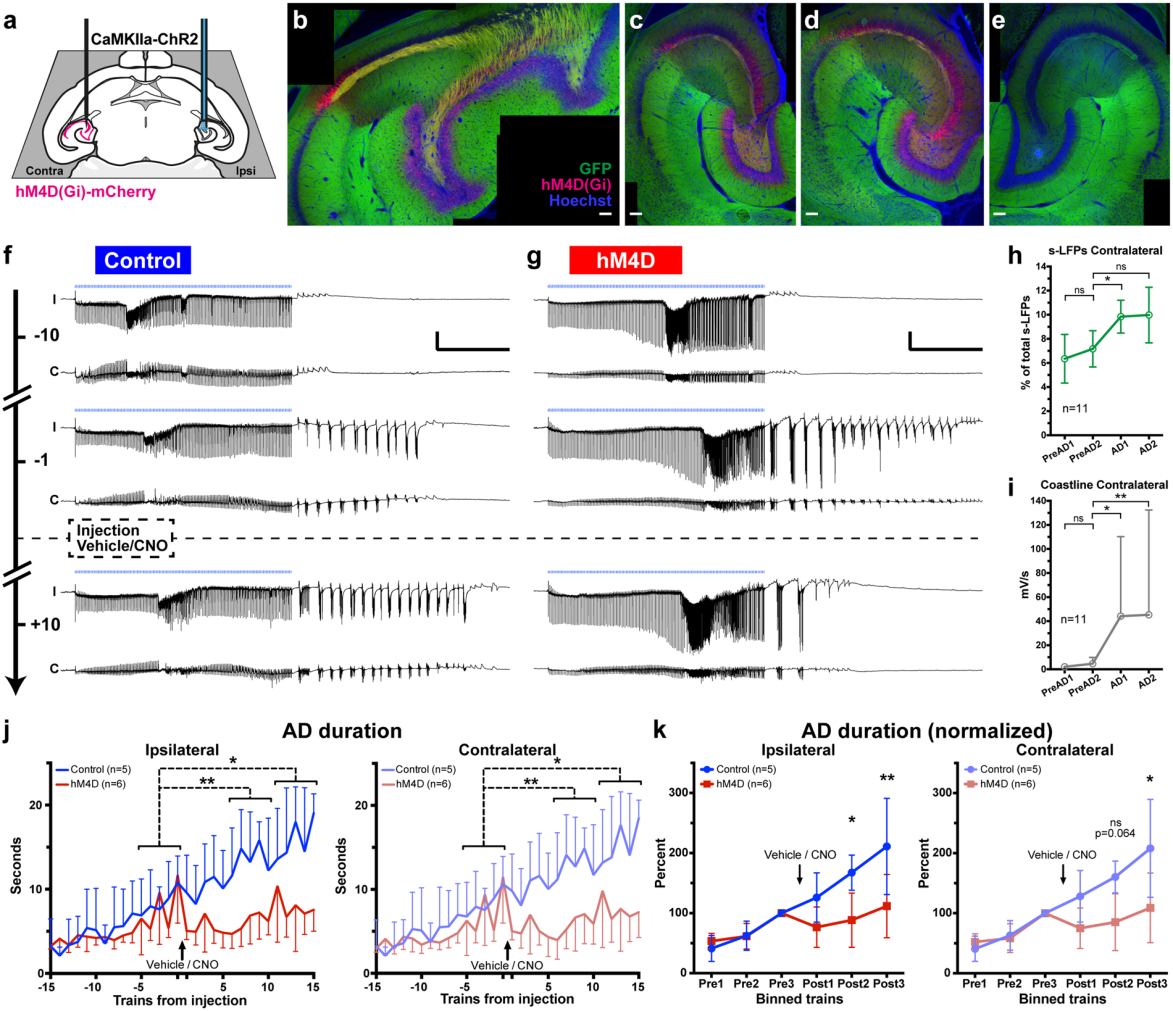
Inhibiting contralateral hippocampus via activation of hM4D blocks AD development induced by optogenetic train stimulation in CaMKIIa-ChR2 mice. (**a**) Illustration of unilateral blue light stimulation with bilateral field recording in horizontal section, targeting ventral CA3/DG at DV-2.1. hM4D(Gi)-mCherry was expressed by AAV vector contralateral to stimulation in the hM4D group. (**b–d**) Representative example of hM4D expression in composite images of horizontal sections from three levels of the hippocampus: dorsal, middle and ventral, respectively. hM4D is visible by mCherry autofluorescence (red; magenta when co-localized with nuclear counterstain (Hoechst, blue)) and ChR2 by YFP (green). (**e**) Representative section of hemisphere ipsilateral to optogenetic stimulation (opposite side of **c**), lacking hM4D expression. Post-experiment burn-in (electrode location) is visible in the hilus. (**f,g**) Traces of bilateral field recordings in control and hM4D mouse, respectively (I: ipsi, C: contra). Timeline arrow indicates number of stimulation trains from IP injection (dashed line) of vehicle or CNO. Bilateral progression of ADs is visibly abrogated after CNO injection in the hM4D-expressing mouse (**g**, bottom). (**h,i**) Counting contralateral intra-train s-LFPs and aligning bins of three stimulations according to the first ipsilateral progressive AD (see Fig. 2f–g), a 37% increase in s-LFPs (**h**, repeated measures ANOVA with Dunnett’s post-hoc test) is seen, coinciding in time with contralateral progressive AD onset (**i**, Friedman test with Dunn’s post-hoc test). (**j**) Quantification of AD durations, which were remarkably similar between ipsilateral (left graph) and contralateral sides (right graph). AD durations did not increase further in hM4D-expressing animals injected with CNO (red line) (repeated measures ANOVA with Dunnett’s post-hoc test, binned data, 5 stimulations per bin (see Results)). (**k**) AD durations of hM4D and control groups in three 5 stimulation bins (Post1-3) covering 75 minutes post-injection were directly compared. Durations were reduced after injection for the hM4D group both ipsi- and contralaterally (left and right graph, respectively) (One-way ANOVA with Sidak’s post-hoc test, see Table 2 for exact P values). Scale bars: (**b–e**) 100 µm; (**f,g**) 5 mV, 5 s. Error bars: ± SD. *P < 0.05, **P < 0.01, ns: non-significant.

Having shown indirect evidence of ADs spreading to the contralateral side in the Thy1-ChR2 mice, by Fos immunoreactivity, we proceeded with an additional set of experiments using bilateral LFP recording, with the aim to obtain direct evidence of ADs appearing in the contralateral hippocampus. Additionally, to ensure exclusive activation of principal excitatory neurons, we used transgenic CaMKIIa-ChR2 mice in these series of experiments, excluding any possibility of co-activation of interneurons by light (see Discussion). Acute ADs were optogenetically induced unilaterally under isoflurane anaesthesia using the same illumination protocol as previously, targeting now mainly the DG and proximal CA3 at the mid-point of the temporal hippocampus, while adding bilateral LFP recording in the same location of the contralateral hippocampal region (Fig. 4a). In these mice the ChR2 expression was particularly strong in dentate gyrus and CA1 (Fig. 4b–e). Again, repeated pulse-trains generated ipsilateral ADs with increasing duration, at the beginning consisting of low amplitude waves, which then developed into higher amplitude, high-frequency progressive ADs (Fig. 4f,g, top and middle “I”-traces, respectively). The high frequency bursts within ADs usually appeared after 15–25 stimulations. Importantly, LFP discharges were detected also in the contralateral hippocampus, although generally with lower amplitude (Fig. 4f,g, “C” traces). These contralateral discharges appeared simultaneously with ipsilateral ADs in a synchronized manner and their durations were very similar bilaterally (see below).

### Contralateral s-LFPs correlate with progressive AD onset

With bilateral recordings, we could now investigate whether there was an increase in contralateral intra-train s-LFPs coinciding with the onset time of progressive AD, much like we had shown for ipsilateral s-LFPs (Fig. 2f,g). Twelve sequential pulse trains around the progressive AD onset were aligned between animals that displayed progressive AD development (n = 11) and contralateral s-LFPs of every three trains were binned, as described above. The number of contralateral s-LFPs increased by 37% in the first bin (AD1) after progressive AD onset (Fig. 4h); 7.2 ± 1.5 vs. 9.8 ± 1.4 (*P* = 0.0058, repeated measures ANOVA (F = 8.3, df = 11) with Dunnett’s post-hoc test: *P* = 0.0138), while a continued trend (10.0 ± 2.3 in AD2) failed to reach significance in the post-hoc test (*P* = 0.073). The marked coastline increase seen ipsilaterally at progressive AD onset (Fig. 2g) was also reflected in contralateral coastline (Fig. 4i): 4.7 ± 5.2 mV/s vs 44.2 ± 66.2 & 45.3 ± 87.1 mV/s (*P* < 0.0001, Friedman test (F stat 25.5) with Dunn’s post-hoc test: *P* = 0.0247 & 0.0029, respectively).

### Contralateral activation of hM4D by CNO halts progressive intensification of ADs

We and others have previously confirmed that neurons expressing the mutated human muscarinic receptor hM4D are hyperpolarized, inhibiting action potentials (APs) *in vitro*^15,22^ and *in vivo*^16^, after addition of clozapine-N-oxide, CNO (most likely acting by subsequent conversion to clozapine, as recently reported)^23^. To test the hypothesis that progressive intensification of ADs induced by optogenetic stimulation in the present study requires contralateral hippocampus, we transduced a subset of CaMKIIa-ChR2 mice with mCherry-tagged hM4D to the hippocampus by unilateral injection of adeno-associated viral (AAV) vector three weeks prior to the experiment (Fig. 4a–d). hM4D expression was confined to the hippocampus contralateral to optogenetic stimulation (Fig. 4c vs e), being strongest in DG and CA3 in the ventral and middle aspects of temporal hippocampus (Fig. 4d,e), and more variable in CA1 and dorsal/septal areas (Fig. 4b).

As previously, progressively intensifying afterdischarges were not achieved in all stimulated animals: ADs fulfilling our electrographic seizure criteria, as judged by continuous monitoring of the post-stimulus periods during recording, were induced in 12 out of 20 CaMKIIa-ChR2 animals in total (one excluded, see Methods), and intraperitoneal (IP) injection of vehicle/CNO was thus performed in 5 control (non-hM4D expressing; 1% DMSO in saline) and 6 hM4D animals (2 mg/kg CNO in 1% DMSO in saline), respectively. In analysis, it was confirmed that the ipsilateral AD durations were similar in both groups averaged for the five stimulations preceding injection: control (n = 5) 8.4 ± 3.6 s, hM4D (n = 6) 7.8 ± 2.9 s (*P* = 0.746, t = 0.33, df = 9, unpaired t-test). Anaesthesia depth, as determined by breathing rate, was not different between mice which did or did not reach progressive ADs, nor between Control and hM4D groups: Progr (n = 11) 63.2 ± 4.1 vs Non-Progr (n = 8) 61.9 ± 3.9 BPM (*P* = 0.4629, unpaired t-test), control (n = 5) 63.9 ± 3.3 vs hM4D (n = 6) 62.7 ± 4.9 BPM (*P* = 0.6283, unpaired t-test).

After IP injections of CNO or vehicle, performed during the 5 minute inter-stimulus period, i.e 2–3 minutes after a stimulation, a further 15 stimulation trains were given over 75 minutes. Sustained progressive intensification of ADs was observed in control animals (Fig. 4f, bottom), similar to our Thy1-ChR2 experiments. The AD durations progressively increased bilaterally and were remarkably similar in length between ipsi- and contralateral sides (Fig. 4j, left & right, respectively). In controls, ipsilateral AD durations increased from 8.43 ± 3.6 s to 13.6 ± 4.2 s post 6–10 and 15.5 ± 2.0 s post 11–15 stimulations (repeated measures ANOVA, *P* = 0.001, F = 17.36, df = 12; Dunnett’s post-hoc test, *P* = 0.004 & 0.013, respectively), while contralateral AD durations increased from 8.35 ± 3.5 s to 12.9 ± 4.1 s post 6–10 and 15.2 ± 1.6 s post 11–15 stimulations (*P* = 0.0035, F = 12.73, df = 12; and *P* = 0.005 & 0.018, respectively); Fig. 4j, blue traces. In contrast, no such increase was seen for the hM4D group ipsilaterally (repeated measures ANOVA, *P* = 0.183, F = 2.10, df = 15) or contralaterally (*P* = 0.195, F = 2.00, df = 15); Fig. 4j, red traces). Effectively, when normalized to pre-injection levels, and binned by 5 stimulations, the average AD duration doubled in controls by the end of experiment while progression of AD duration in hM4D-expressing animals was significantly halted after CNO injection compared to controls (One-way ANOVA with Sidak’s post-hoc test, see Table 2 and Fig. 4k). Importantly, anaesthesia depth was not contributing to these differences, as breathing rates remained unchanged throughout the experiment in both vehicle-injected controls and CNO-injected hM4D animals (Supplementary Fig. S2). In addition, there was no indication of compensatory reduction of isoflurane, as levels were unchanged in the hM4D group by the end of experiment. Thus, sedative effects by accumulation of converted clozapine^23^, are very unlikely given the administered dose of CNO and timespan of the experiment (see further in Discussion).

**Table 2:** Binned values for AD metrics post-injection of CaMKIIa-ChR2 mice, normalized to pre-injection levels.

	ANOVA	F stat.	Normalized level to pre-injection (% ±SD)					
			Post 1 (0–25 min)		Post 2 (25–50min)		Post 3 (50–75min)	
			Control	hM4D	Control	hM4D	Control	hM4D
AD duration	Ipsi		126 ± 41.0	76.6 ± 33.6	168 ± 29.2	88.3 ± 45.1	211 ± 80.1	112 ± 52.7
	*P* = 0.0010	5.679	*P* = 0.29		*P* = 0.039*		*P* = 0.0076**	
	Contra		128 ± 42.9	75.0 ± 33.7	160 ± 26.4	85.1 ± 47.2	208 ± 81.5	109 ± 58.0
	*P* = 0.0020	5.129	*P* = 0.26		*P* = 0.063		*P* = 0.010*	
Max amplitude	Ipsi		116 ± 29.5	84.9 ± 27.2	233 ± 73.9	90.2 ± 28.0	205 ± 105	96.0 ± 21.1
	*P* = 0.0002	7.537	*P* = 0.74		*P* = 0.0005***		*P* = 0.0075**	
	Contra		95.0 ± 53.7	97.5 ± 11.7	88.9 ± 73.2	105 ± 31.9	103 ± 59.0	98.5 ± 24.7
	*P* = 0.99	0.085	*P* > 0.99		*P* = 0.92		*P* > 0.99	
AD coastline	Ipsi		190 ± 147	72.8 ± 60.2	403 ± 234	99.4 ± 73.7	409 ± 197	146 ± 98.0
	*P* = 0.0009	5.827	*P* = 0.46		*P* = 0.0049**		*P* = 0.016*	
	Contra		130 ± 102	71.9 ± 50.0	160 ± 155	108 ± 86.9	191 ± 117	151 ± 123
	*P* = 0.54	0.824	*P* = 0.77		*P* = 0.82		*P* = 0.91	

Control (n = 5), hM4D (n = 6). Values are binned from five successive stimulations, post injection. Overall significance levels and F statistic by one-way ANOVA is given in the 2nd and 3rd columns. Significance in Sidak’s post test (comparing control vs hM4D for each bin pairwise) is denoted with P-values underneath each pair. *P < 0.05, **P < 0.01, ***P < 0.001.

Together, these data suggest that physiological inhibition of contralateral hippocampus by temporarily hyperpolarizing hM4D-expressing neurons, without structurally severing the interhemispheric axonal connections, was sufficient to counteract the progressive intensification of ADs.

### AD burst characteristics were not different between hM4D-expressing and control CaMKIIa-ChR2 mice, and were not affected by contralateral inhibition

**Figure 5:**
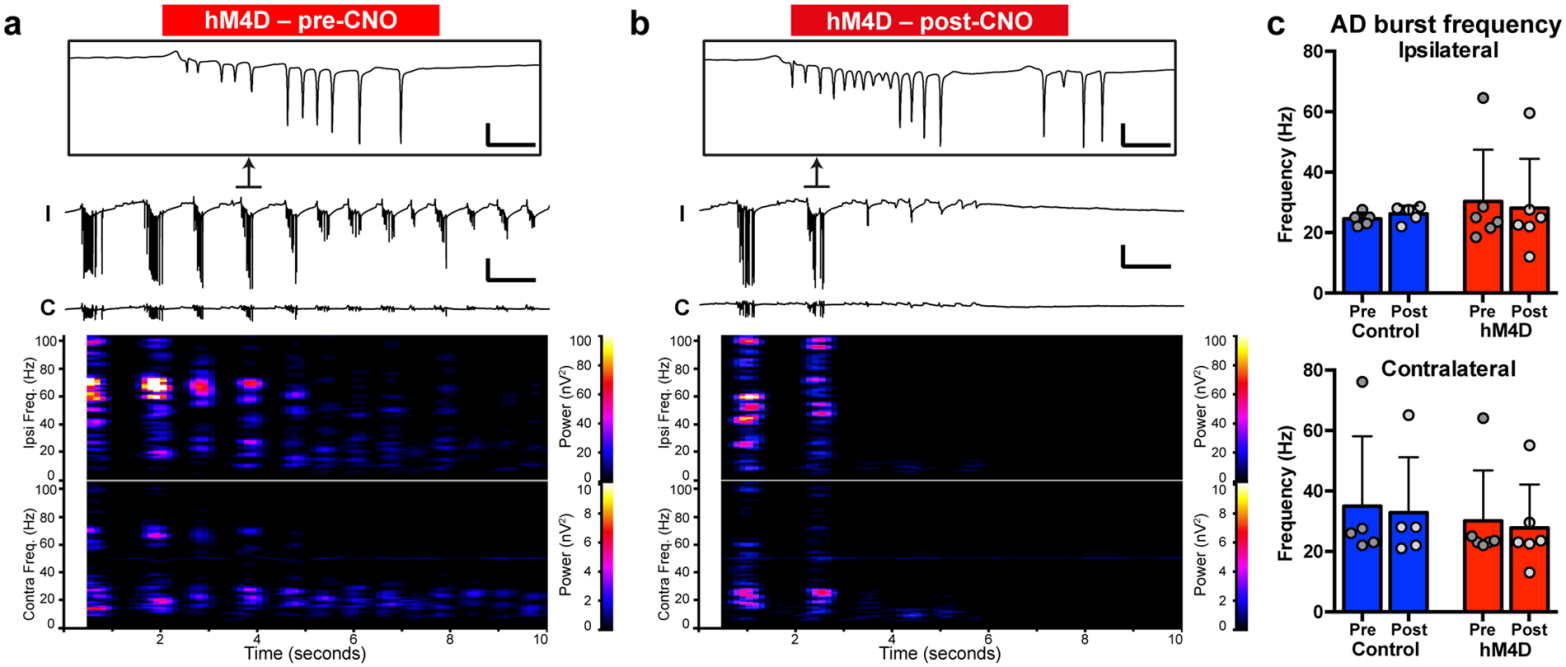
Afterdischarge burst frequencies are not affected by inhibition of contralateral hippocampus. (**a,b**) 10 s post-stimulation periods immediately preceding CNO injection (**a**) and 10 stimulations post CNO injection (**b**), with details of ipsilateral bursts at the top (same animal as in Fig. 4g). Recording traces were Fast Fourier Transformed to examine burst frequencies (bottom). AD burst power was concentrated to frequency ranges of 15–30 Hz and 60–90 Hz in both groups. (**c**) Quantification of the burst frequencies on group level, taking the mean of the median burst frequency for each animal. There was no difference between groups (one way ANOVA), or from pre- to post-injection within groups (paired t-test), either ipsilateral or contralateral (see Results for *P* values). Scale bars (**a & b**): box: 5 mV, 50 ms; 5 mV, 1 s. Error bars: ± SD.

To further investigate whether hM4D activation had an impact on AD characteristics itself, frequency analysis was performed on 15 second post-stimulation periods by Fast Fourier Transformation, after downsampling to allow for 1 Hz resolution in a 1 second window. High frequency bursts were largely stratified in two clusters of either 15–30 Hz or 60–90 Hz (Fig. 5a,b) in both control and hM4D animals, but overall 25 Hz was dominating (Fig. 5c). Examples of bursts are magnified in Fig. 5a,b, top. When making group comparisons, the median burst frequency from each animal was used before averaging. The burst frequencies did not differ between groups (One-way ANOVA, Ipsi: *P* = 0.886, F = 0.213, df = 18; Contra: *P* = 0.920, F = 0.163, df = 18) or pre- to post-IP injection (paired t-test Ipsilateral: *P* = 0.364 & 0.523, t = 1.02 & 0.687, df = 4 & 5, Control/hM4D, respectively; Contralateral: *P* = 0.411 & 0.400, t = 0.917 & 0.919, df = 4 & 5, respectively); ipsilateral frequencies (Fig. 5c, top): control pre- and post-IP injection 24.5 ± 2.1 & 26.2 ± 2.7 Hz; hM4D pre- and post-IP injection 30.3 ± 17.1 & 28.1 ± 16.3 Hz; contralateral frequencies (Fig. 5c, bottom): control pre- and post-IP injection 35.0 ± 23.1 & 32.9 ± 18.3 Hz; hM4D pre- and post-IP injection 30.1 ± 16.7 & 27.8 ± 14.4 Hz. These data suggest that the control and hM4D groups did not differ in regards to AD phenomenology prior to injection, and also shows that contralateral inhibition by CNO did not change the frequency of bursts in the hM4D group, although the progression of ADs was halted (see previous section).

## Discussion

We demonstrate that selective optogenetic activation of principal neurons in the hippocampus leads to an increase in both light-induced and spontaneous LFPs over the course of successive light pulse-train stimulations. Observing such plastic changes during pulsed stimulation has become possible due to optogenetics, circumventing the occlusion of the LFP by the electrical stimulations, as was normally the case in previous studies. This increase in LFPs, suggesting progressive elevation of excitability in both the local and contralateral network, was time-matched by the progressive intensification of ADs. In addition, progressive ADs were effectively halted by temporary functional inhibition of contralateral hippocampal neurons by chemogenetics (inhibitory DREADD hM4D), suggesting the necessity of transhemispheric hippocampal involvement in this process.

**Figure 6:**
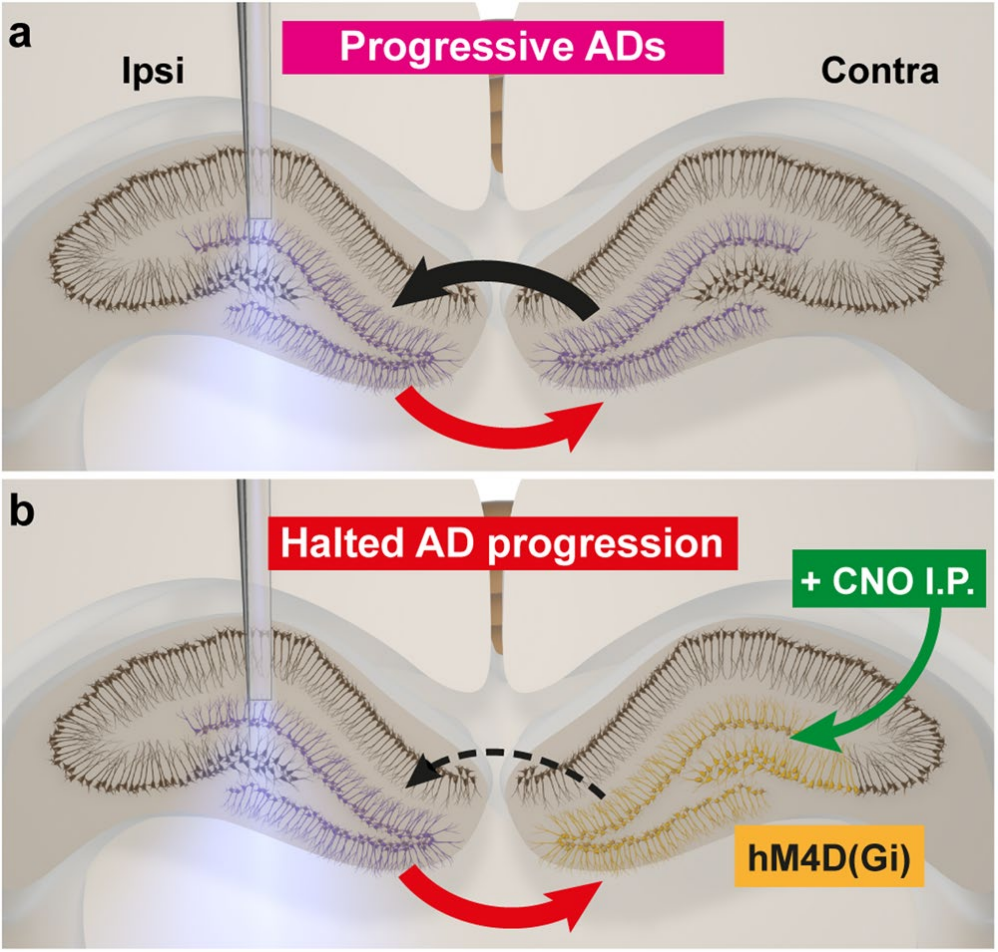
Schematic illustration of proposed mechanism for transhemispheric loop required for hippocampal progressive ADs. (**a**) Repetitive unilateral optogenetic stimulation leads to progressive increase in excitability of the local network, as evidenced by increased number of spontaneous LFPs during the stimulation train (ipsilateral s-LFPs, Fig. 2). These spontaneous LFPs spread to the contralateral hippocampus (red arrow), activating the contralateral dentate gyrus (contralateral s-LFPs, Fig. 4h), thus creating a transhemispheric feedback loop (black arrow) leading to progressive intensification of ADs bilaterally. Purple cells denotes increased activity indicated by Fos or LFP recordings. (**b**) If the contralateral hippocampus is inhibited, such as via hM4D-expressing neurons (located mainly in DG and CA3, orange) of CaMKIIa-ChR2 animals by CNO IP injection (green), feed-forward still exists (red arrow), while the feedback loop is selectively and temporarily disrupted (dashed arrow) and progressive intensification of ADs is halted.

Our optogenetic approach enabled a localized activation and recording of principal cell populations, which demonstrated that in order for progressive intensification of ADs to occur, the local network needs to be modified so that extra spontaneous LFPs are generated: such LFPs were almost exclusively seen in animals displaying rapid progression of high-amplitude ADs (Fig. 2) and bilateral DGC Fos reactivity (Fig. 3). With bilateral recording, we recorded s-LFPs on the contralateral side during stimulation, with a typical pattern of bilateral transhemispheric synchronization (Fig. 4f,g), for both LFPs and ADs. Interestingly, the recruitment of contralateral hippocampus into hypersynchronized AD generation also coincides in time with an increase in numbers of s-LFPs during the stimulation train (Fig. 4h,i), suggesting that not only the local network recruitment, but also the synchronous generation of transhemispheric LFPs, is needed to reach a presumed threshold when progressive AD intensification can be initiated (see Fig. 6a). Only in this case does the overall hippocampal network become more excitable, triggering plastic changes leading to progressive hypersynchronized activity.

Progressive afterdischarges induced by optogenetic stimulations in the present study (Figs 1 & 4) are phenomenologically similar to the rapid kindling observed with electrical stimulation of the hippocampus *in vivo*^6,24,25^. Moreover, optogenetic stimulation resulting in kindling-like seizure development has been reported in freely moving mice selectively expressing ChR2 in DGCs^12^. In contrast, this progressive nature of optogenetically induced ADs was not observed in another earlier publication in rats^13^. This discrepancy could be due to a different site of stimulation and species, but more likely relates to the differences in power of light used for stimulations (up to 30 times higher in Osawa *et al*. compared to the present study), which may have activated much larger populations of neurons. In the present study, with lower light intensity, a smaller tissue volume is expected to be activated. This may have resulted in the observed diversity in stimulation outcomes due to variability in ChR2 expression respective to optrode positioning. It should also be noted that hippocampal ChR2 expression in the Thy1-ChR2 (strain 18) mice may not be entirely limited to principal neurons. Interneuron expression cannot be ruled out because of the nature of the Thy1.2 cassette^26,27^. Indeed, ChR2 expression in interneurons has been reported, particularly in juvenile animals^28^, but also in adults (although in another sub-strain)^29^, and we have also found evidence supporting this notion (see Supplementary Fig. S3). It is possible that the direct optogenetic activation of interneurons in the small volume of illuminated tissue would inhibit local network and thereby prevent bilateralization of the dentate activation. In CaMKIIa-ChR2 mice, where ChR2 expression is exclusive to the principal excitatory neurons, light-induced APs only indirectly activate interneurons, phase-locking their activity with network oscillations. Therefore, the interneurons are more likely to contribute to synchronization and generation of ADs^30–32^, rather than AD inhibition. This may explain why intensification of ADs was observed more often (in 12 out of 20 mice, as compared to 4 out of 21). However, we cannot exclude that differences in the specific ChR2 expression patterning between Thy1-ChR2 and CaMKIIa-ChR2 mice together with the slightly adjusted targeting of the inserted optrode also contributed to this observation.

By hyperpolarizing hM4D-expressing neurons in the contralateral hippocampus after injection of CNO (Fig. 6b), we demonstrate that a transhemispheric network, possibly connected via CA3 neurons^33,34^ or hilar mossy cells^35,36^ projecting across the hippocampal commissures, is necessary for progressive intensification of ADs. AD durations were significantly reduced bilaterally after CNO injection (Fig. 4j,k) compared to the controls, while the two groups were comparable prior to injection (Figs 4j & 5). Compared to AD amplitude and frequency, the AD duration is the most important parameter, since it is less sensitive to the exact location of the recording electrode (i.e. vicinity of a cell layer). Overall, our findings gain support from previous studies with electrical stimulation of the hippocampus (in rats) where maximal dentate ativation and bilateralization were found to be required for kindling^37,38^. However, previous studies performed complete severing of transhemispheric axonal connections to show bilateral dependency, which may lead to reorganization of both local and remote networks with potentially unknown consequences for the whole network system. In contrast, we used only temporary inhibition of the contralateral hippocampus by chemogenetics, thereby significantly substantiating earlier findings by excluding the potential cofounding effect of permanent lesions in the commissural pathway.

Activation of hM4D expressed in the focus of seizure induction has recently been shown to inhibit both acute and chronic cortical seizures in animal models^39^. Likewise, local hippocampal optogenetic intervention has been shown to suppress spontaneous seizures in a TLE model^40^. In addition, spontaneous seizures can be inhibited by intervention with remote network components demonstrated in different seizure and epilepsy models using optogenetic^32,41–43^ or chemogenetic^44^ approaches ipsilaterally. In the present study however, we demonstrate that progressive intensification of ADs can be halted by inhibiting neuronal activity by hM4D in a transhemispherically located remote component of the network, in the contralateral hippocampus. Our data therefore suggests that temporary functional inhibition of critical remote network components of seizure-generating circuitry may be sufficient to modify progressive intensification of ADs. It still remains to be established whether hM4D-based inhibition of other transhemispheric network components (e.g. amygdala, etc.) may also exert a similar inhibitory effect, or that optogenetic stimulation of such areas might also activate the dentate gyrus.

It was recently demonstrated (after completing the current experiments) that DREADD receptors in the CNS *in vivo* are activated by clozapine converted from CNO, rather than by CNO itself^23^. Clozapine could then potentially exert endogenous effects on various cellular and neurotransmitter systems as an antipsychotic drug, e.g. sedation^23,45^. However, several factors in our experimental set-up would render this unlikely: the concentration of clozapine resulting from CNO conversion is far below the threshold for endogenous CNS effects (0.04 mg/kg at 2% CNO conversion, compared to 1 mg/kg for sedative effects shown in awake mice). Also, accumulation of converted clozapine is not likely to contribute, as our trial period was only 75 minutes, compared to the 2 hours needed for sedative signs in Gomez *et al*. Moreover, previously described effects on the dopamine system are very unlikely, again due to low CNO concentration (dopaminergic effects were not seen at 2 mg/kg CNO)^45^. Likewise, effects on the serotonergic system should be negligible since our mice were already under isoflurane anaesthesia, greatly reducing serotonin levels^46^. Any additional potential 5-HT receptor blockade by clozapine (present only at sub-threshold levels already) are thus not expected to have an impact. In conclusion, together with our data on anaesthesia and isoflurane levels (see Results), there is no reason to believe that converted clozapine affected the outcome and/or interpretation of our experiments.

A recent study showed that progressive seizures could be induced by optogenetic stimulation of the DG in awake mice^12^. However, inhibition of the contralateral DG by optogenetic activation of NpHR had no effect on spontaneous seizures in chronically epileptic mice. Taken together with our data, this suggests that inhibition of the contralateral hippocampus may be sufficient to halt progressive intensification of ADs at an early stage of kindling, but is no longer effective in counteracting spontaneous seizures in chronic epilepsy models, where permanent network reorganization, such as e.g. mossy fiber sprouting, has already occurred^47–49^.

In conclusion, the selective and minimal optogenetic activation of hippocampal circuitry is a useful new tool for probing early changes in network plasticity leading to hyperexcitability and progressive intensification of ADs. Moreover, the data provides direct evidence that selective repetitive activation of principal neurons induces increased excitability of initially local followed by remote (transhemispheric) hippocampal network components, and that their cross-interaction is necessary for progressive intensification of ADs. Our data encourages further investigations on the transhemispheric network mechanisms of seizure progression in the temporal lobes, which may reveal intriguing possibilities for selectively targeting the hippocampus contralateral to the emerging seizure focus to prevent progression and even development of chronic TLE.

## Methods

### Animals, surgery and chemicals

Animals were bred at the local animal facility and kept in 12 h light/dark cycle with access to food and water ad libitum. All procedures were approved by the Malmö/Lund Animal Research Ethics Board, permits M206-12 & M47-15, and were carried out in accordance with local guidelines and regulations. First, 33 (24 female) Thy1-ChR2-YFP mice (strain#18, Jackson #007612), 6–15 months old, were used (including nine controls). Three were excluded (prior to analysis due to technical errors). For the second series of experiments, we generated CaMKII-ChR2 mice by crossing the T29-1 (Camk2a-cre) and Ai32 (RCL-ChR2/eYFP) strains (Jackson, #005359 & #012569, BL6 background). Offspring was genotyped for ChR2-YFP fusion protein with a PTC200 PCR machine (BioRad). 20 mice (8 females) 4–9 months old were used, divided into hM4D-injected (n = 13, see below) and control (n = 7) groups. Nine animals were excluded: eight (2 control, 6 hM4D) did not reach AD progression criteria (see further below) and one additional progressive hM4D animal died after failed IP injection. Estrous cycle of female mice was not monitored. For surgery, Isoflurane (Baxter) anaesthesia was maintained at ~2.5% (4% at induction) in 100% oxygen, with bupivacaine (Marcain, AstraZeneca) as local analgesic. In brief, mice were anaesthetized and placed in a small animal stereotax (Kopf), with electric or water-heated pads to maintain body temperature. A midline incision was made and connective tissue removed, bregma and lambda were aligned using a blunt reference rod. A small hole was drilled at defined coordinates (relative to bregma), bone pieces removed by fine forceps and dura was punctured with a hypodermic needle. Physiological saline (0.5 mL) was injected subcutaneously to prevent de-hydration, and chlorhexidine (Fresenius Kabi) was used for wound cleaning. Sigma or VWR supplied all stock chemicals except where stated otherwise.

### Viral vector injection

Thirteen CaMKII-ChR2 mice, 3–7 months old, were anaesthetized and stereotactically injected with AAV8-hSyn-hM4D(Gi)-mCherry (UNC vector core, titer 7.4 × 10^12^) into the left ventral hippocampus at AP-3.2, ML-3.1 using pulled glass capillaries (Stoelting, #50811) fitted to a 5 µL 75RN-26S syringe with 22 S 51 mm needle (Hamilton) with deposits made at DV (from brain surface) −3.6 and −3.2 (0.5 µL each, 0.1 µL/min), waiting for 5 minutes after each deposit to avoid back-flow. The wound was closed with Histoacryl tissue glue (B. Braun) and sutured with resorbing thread (Ethicon).

### *In vivo* recording setup

Mice were anaesthetized and placed in a stereotax. Coordinates were AP-2.6, ML-3.1 for unilateral recording with Thy1-ChR2 mice targeting the ventral CA3 of the right hippocampus at DV-2.75 mm (DV-3.2 from bregma, see sagittal plate 127^50^), while bilateral recording in CaMKII-ChR2 mice, was performed at AP-3.2, ML ± 2.6 targeting the middle portion of ventral CA3/DG bilaterally at DV-2.1. Before starting the recording session, isoflurane was lowered and incrementally adjusted throughout recording to maintain a breathing rate of 50–70 BPM, to standardize anaesthesia depth across the animals. Sufficient anaesthesia was controlled by toe pinch. Average isoflurane level for Thy1-ChR2 was 1.47 ± 0.12% (males) and 1.43 ± 0.18% (females) and for CaMKII-ChR2 1.73 ± 0.38% (males) and 1.42 ± 0.14% (females). There was no sex difference in anaesthesia depth, as evaluated by breathing rate: Thy1-ChR2 M (n = 3) 53.6 ± 5.2 vs F (n = 18) 52.8 ± 7.1 BPM (*P* = 0.8648), CaMKII-ChR2 M (n = 12) 62.9 ± 4.3 vs F (n = 8) 62.2 ± 3.2 BPM (*P* = 0.711, unpaired t-tests). An optrode consisting of a 200 µm diameter optic fiber (FT200EMT, Thorlabs) with flat-cleaved end glued to a tungsten electrode (TM21A10, WPI UK), with the electrode tip spaced 0.5 mm from the fiber end, was lowered into the right hemisphere using a MO-10 micromanipulator (Narishige). For bilateral recording, an additional tungsten electrode medio-laterally spaced 5.2–5.4 mm from the optrode, was attached to the micromanipulator. LFPs were recorded via extracellular amplifiers (DP-311, Harvard apparatus) with 10-times amplification and 0.1–3k Hz bandpass filter, and digitized at 10 kHz on a PowerLab 4/35 (AD Instruments) connected to a Windows PC with LabChart Pro (AD Instruments). A copper-mesh cage blocked electro-magnetic interference. The optic fiber connected to a 463 nm blue LED (Prizmatix), controlled via the PowerLab. Prior to each experiment the metered light power output (1916-C, Newport) was adjusted to 2.9 ± 0.2 and 2.6 ± 0.1 mW (Thy1-ChR2 and CaMKII-ChR2, respectively), corresponding to a maximal output at the fibre tip of 92 mW mm^−2^, which should not harm the tissue^11^. Effective illumination volume was calculated, approximated as a sphere^51^, using the irradiance distance calculator of the Deisseroth lab webpage (web.stanford.edu/group/dlab, based on actual measurements in brain tissue), and considering 1 mW mm^−2^ the lower bound for ChR2 activation^52^. The optrode ensemble was, after initial insertion and resting for 5 minutes at a depth of DV-1.5 mm, lowered in 50–100 µm increments with 30 s intervals, giving two 250 ms light pulses each time, probing the maximal field response, indicating vicinity of a cell layer. For bilateral recordings, contralateral responses were also considered. If the test-pulse responses decreased at further depths, the optrode was maximally retracted 0.5 mm, to not record from tissue damaged by the optrode. Final depths for recording were DV-2.9 ± 0.46 mm (Thy1-ChR2), and DV-2.0 ± 0.33 & DV-2.1 ± 0.21 (CaMKII-ChR2 hM4D & control groups, respectively). Note that the photovoltaic effect reported by others^11^ did not account for our recorded responses: in measurements of ChR2-negative mice (data not shown) the amplitude of such artifacts were in the µV range, 100-fold lower than the amplitudes of the above mentioned LFPs.

### Optogenetic train stimulation

10 Hz trains of 5 ms blue light pulses were 15 second long (i.e. 5% duty cycle, 150 pulses per train). In Thy1-ChR2 animals, pulse-trains were repeated 40 times, with 5-minute inter-train interval (Fig. 1c), total recording period ~3.5 hours. Thy1-ChR2 stimulation controls (n = 3) were subjected to similar treatment, except blue light was only given for pulse-trains 1, 20 and 36–40, the remainder consisted of 593 nm laser light pulses (CrystalLaser) at the same power, i.e. in total the control animals were given 33 yellow and 7 blue light pulse-trains. A Thy1-ChR2 Fos control group (n = 6) were given a single blue-light stimulation train, followed by perfusion 15 minutes later. Thy1-ChR2 mice later classified as Progressive or Non-Progressive (see Results) had the following sex distribution: Progr: 2 M, 2 F; Non-Progr: 1 M, 16 F.

### Optogenetic train stimulation with CNO injection

Pulse-trains were applied to CaMKII-ChR2 mice, minimum three weeks after viral vector injection, as described above except repeated until ADs containing oscillatory bursts appeared ipsilaterally each time after five consecutive stimulations, with average AD durations ≥5 seconds. If an animal did not reach this electrographic seizure progression criterion (based on the phenomenology of Thy1-ChR2 progressive group) within 35 stimulations, recording was aborted and the animal was excluded (n = 8, 2 male, 6 female). Then 10 µL/gram bodyweight vehicle (1% DMSO in saline) or Clozapine-N-Oxide (Tocris, 0.2 mg/mL in 1% DMSO-saline; i.e. 2 mg/kg) was injected IP (control and hM4D group, respectively, total n = 12, 10 male, 2 female (one excluded as explained previously)), followed by 15 additional stimulations (75 minute time-span). An electrode position “burn-in” was applied with three 500 µA 1 s pulses (DS3 stimulator, Digitimer) to aid post-mortem recording site localization.

### Immunohistochemistry

Animals were given a sodium pentobarbital IP injection (40 mg/kg) and were transcardially perfused with ice-cold 0.9% saline followed by 4% PFA in 0.1 M phosphate buffer (PB). Total time from the last stimulation to perfusion was approximately 15 minutes. Brains were post-fixed in PFA overnight before transfer to 20% sucrose in PB. After ≥24 h brains were cut in horizontal 30 µm slices on a microtome (Thermo Scientific) and stored in an ethyleneglycol/glycerol-based antifreeze. For Fos staining, sections were rinsed in potassium PBS (KPBS) followed by KPBS with 0.25% Triton X-100 (T-KPBS), then blocked in 5% Normal Donkey Serum (Millipore) in T-KPBS and incubated with 1:500 primary Goat-anti-cFos polyclonal antibody (Santa Cruz Biotechnology, sc-52-g) overnight. Sections were rinsed and incubated with 1:300 Biotin-SP-conjugated Donkey-anti-goat followed by 1:200 Cy5-streptavidin for 2 hours each (Jackson Immunoresearch, 705-065-147 & 016-130-084, respectively), then rinsed and mounted on coated glass slides and coverslipped with 2.5% PVA-DABCO mounting medium containing 1:1000 Hoechst-33258 or Hoechst-33342 solution (Invitrogen/Molecular Probes/Life Technologies, H3569/H3570) for nuclear counterstain. Slices from CaMKIIa-ChR2 mice were similarly mounted after being rinsed 3 times in KPBS.

### Image acquisition and analysis

Images were acquired on a BX61 microscope with a DP72 CCD camera (Olympus) in “.vsi” format using CellSense Dimension software (Olympus), with separate fluorescence-filtered channels as 8-bit monochrome images, from four different parts of the hippocampus in each hemisphere (see Fig. 3h). The same contrast settings were used for all slides from the same brain. The pictures were imported to Fiji (ImageJ2, 2.0.0-rc-14/1.49 g), converted to Tiff and stitched together using the plugin described in Preibisch *et al*.^53^. For Fos intensity analysis, stitched images of the DG at 20 times magnification were used. In brief, regions of interest (ROI) delineating the DGC were determined from nuclear stain images. Cy5 (cFos) background level was measured from the uniformly stained outer rim of the DG molecular layer. Cy5 mean pixel intensities were then measured by overlaying the DGC ROIs, and finally normalized by adding the mean background of all brains subtracted by the background of the section. Animals with overall higher intensities bilaterally compared to the control group (average of all four regions) were assigned as “Bilaterally activated” (BiL, n = 4). Those with higher cFos levels in at least one area ipsilateral (to stimulation) than the control, and overall ipsi- to contra-lateral ratio of more than 2 standard deviations of control (>128%) were assigned as “Ipsilaterally activated” (IpL, n = 10), the remainder were assigned as “Non-activated” (NA, n = 7).

### Analysis of field recordings

Field recordings were processed and analyzed in LabChart (AD Instruments) and Excel (Microsoft), and visualized in Igor Pro 6.37 (WaveMetrics), notch filtered at 50 Hz. Coastline (CL) was calculated on the 0.5–300 Hz bandpassed signal as the absolute voltage difference between every other sample, integrated over the first five seconds after each stimulation train and divided by time. Baseline CL/s (i.e. noise) was subtracted. Afterdischarge duration was measured manually as the time from the end of stimulation to the last peak where coastline was ≥5 times baseline RMS, while represented on the recording as a clear peak or wave. Due to differences in noise level, detection threshold varied by animal. Intra-train LFPs were counted using the slope (derivative) of the recording, smoothed over 1 ms. Detection threshold was set separately for each animal, adjusted to avoid false positives (minimum was 250 mV/s). Post-light i-LFPs were defined as events occurring from 3 ms after the start of the light pulse (avoiding the direct light activation peak) until the end of the slow wave LFP response (possibly synaptic, 25–30 ms), while s-LFPs were defined as those detected up until the next light pulse (Fig. 2a). Peak properties of s-LFPs were extracted from the last 10 s of the first stimulation train displaying high frequency ADs, using the Peak Analysis plug-in with automatic baseline detection. LFP rise time, decay time & amplitude was calculated from the 10–90% range of detected peak heights, LFP width at 50% of the peak height. Frequency analysis was performed in LabChart by resampling a 7 Hz high-pass filtered signal to 256.4 samples/s, followed by Fast Fourier Transformation with a 256-sample Hann window and overlap set to 87.5%. At least one representative burst from each post-stimulation AD containing high-frequency bursts was selected for measurement, at least 0.5 s away from the end of stimulation to avoid 10 Hz artifact overlap. The maximum power frequency was then extracted from the selection in the 8–90 Hz band.

### Data analysis and statistics

Data is presented as mean ± standard deviation (SD) except for non-normally distributed data where medians and 95% confidence intervals (CI) or 25–75% percentiles are given. Whiskers in all graphs represent SD, except boxplots (min/max). Statistical analysis and graphing was done in Prism 6.0e (GraphPad) with alpha level of 0.05 and two-tailed tests used throughout. Parametric or non-parametric tests were chosen based on the Shapiro-Wilk normality test. Comparing two groups, Student’s t-test was used. For three groups, one-way ANOVA with Dunnett’s or Sidak’s post-hoc test, or Kruskal-Wallis test with Dunn’s post-hoc test was used. For repeated measures, paired Student’s t-test, repeated measures ANOVA with Dunnett’s post-hoc test or Friedman test with Dunn’s post-hoc test was used. For CaMKIIa-ChR2 mice AD data, five stimulations were binned, aligning bins to the time-point of vehicle/CNO IP injection. For post-injection pairwise comparison, data was normalized to the bin prior to IP injection (“Pre3”) for each animal.

### Data availability

The datasets generated and/or analysed during the current study are available from the corresponding author on reasonable request.

## Electronic supplementary material

Supplementary Information
