# Supplementary material for: Dynamic interaction of local and transhemispheric networks is necessary for progressive intensification of hippocampal seizures
Source: Sci Rep. 2018 Apr 4;8:5669. doi: 10.1038/s41598-018-23659-x (PMC5884800; doi:10.1038/s41598-018-23659-x)
Supplement: Supplementary file 1 — Supplementary Information [file 41598_2018_23659_MOESM1_ESM.pdf]

Supplementary Information

Dynamic interaction of local and transhemispheric networks is necessary for progressive intensification of hippocampal seizures

Fredrik Berglind, My Andersson, Merab Kokaia

Epilepsy Centre, Dept of Clinical Sciences, Lund University, Sweden
Corresponding author: merab.kokaia@med.lu.se

Supplemental Methods

Breathing rates and isoflurane levels

Breathing rate was recorded by an MLT1010 pulse transducer (AD Instruments) positioned under the ribcage of the mouse, connected to the PowerLab 4/35 (AD Instruments), and instant BPM was calculated by the cyclic measurements tool (LabChart). The mean overall BPM was averaged over the final 25 stimulations given (~120 minutes). For BPM comparisons in the CaMKII-ChR2 groups (hM4D vs Control), the mean was calculated over the 90 second periods prior to a stimulation pulse-train and then binned per 5 stimulations, covering 25 stimulations (2 bins before and 3 bins after injection). hM4D and Control BPMs were compared in a 2-way ANOVA with Dunnett's post-hoc test (GraphPad Prism) with time as a row factor and treatment as column factor. Isoflurane percentage levels noted in the recording lab books were averaged, weighted for the time spent at each level, and analysed by repeated measures ANOVA with Dunnett's post-hoc test and, normalized for the pre-injection bin (Pre2), by one way ANOVA with Sidak's post-hoc test.

Intra-train LFP frequency patterns

Detection of sLFP lag and LFP frequency patterns was performed on the same pulse-trains as used for s-LFP property evaluation (Fig 2h), i.e. the train at which progressive ADs started. The lag was calculated by subtracting the time of the preceding light pulse with that of the first detected s-LFP for each inter-pulse period. Rhythmic LFP frequency was calculated as the median of detected LFP rates (in Hz, using the LabChart cyclic measurement tool) in each of the 0.1 second inter-pulse periods (i-LFPs and s-LFPs were not distinguished). By taking the median, temporary variations (maximum and minimum) of LFP frequencies (due to the presence of direct 10 Hz light-induced LFPs (L-LFP), or gaps in appearance of s-LFPs) were thus minimized. Peak (maxima) and mean rhythmic LFP frequency was extracted from continous sections exceeding 20 Hz, corresponding to rhythmic LFP activity.

PV-interneuron staining and confocal imaging

Histological sections from Thy1-ChR2 mice were prepared as described in main Methods, except primary was 1:1000 polyclonal Rabbit anti-PV (Abcam, ab11427) and secondary 1:400 Cy3-Donkey-anti-Rabbit (Jackson Immunoresearch, 711-165-152) antibodies. Confocal images were acquired on an LSM710 NLO laser scanning microscope (Zeiss) fitted with an Achroplan 40x, 0.8 NA water-immersion objective (Zeiss), z-stacks at 1.24 µm inter-plane distance. Illumination and detection wavelengths were 488 nm, 493-543 nm (GFP) and 543 nm, 548-681 nm (Cy3), respectively. Images were assembled in ImageJ2, and auto-leveled for purposes of clarified display only.

Supplementary Figures & Figure Legends

Figure S1 (of 3)

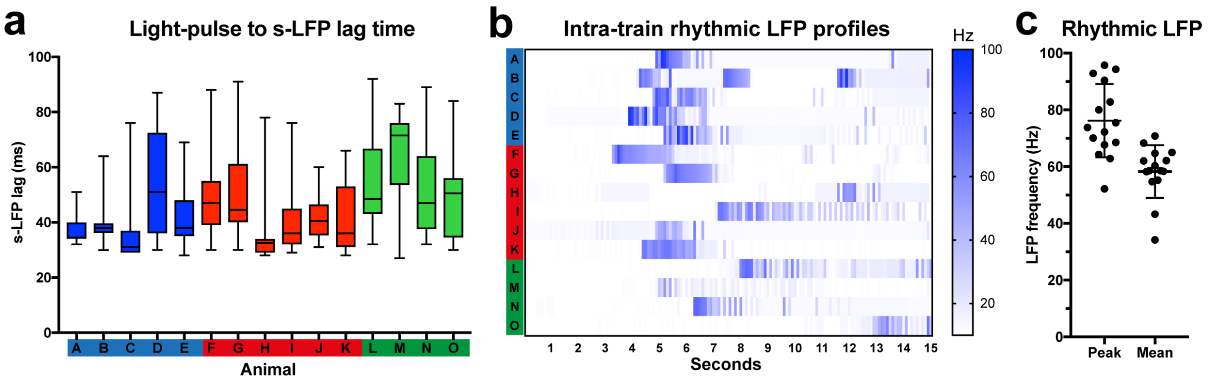

Figure S1. Intra-train s-LFP lag and rhythmic properties in mice with progressive AD phenomenology. **A** The measured average lag time from light pulse to first s-LFP in one stimulation train from each animal, corresponding to the start of progressive post-stimulation AD generation for each animal. Blue: CaMKII-ChR2 controls, Red: CaMKII-ChR2 hM4D, Green: Progressive Thy1-ChR2 group. **B** Median rhythmic properties of intra-train LFPs extracted from the same stimulation trains as in A. **C** Peak and mean frequency of the main continous section of rhythmic LFPs for each animal in B.

Figure S2 (of 3)

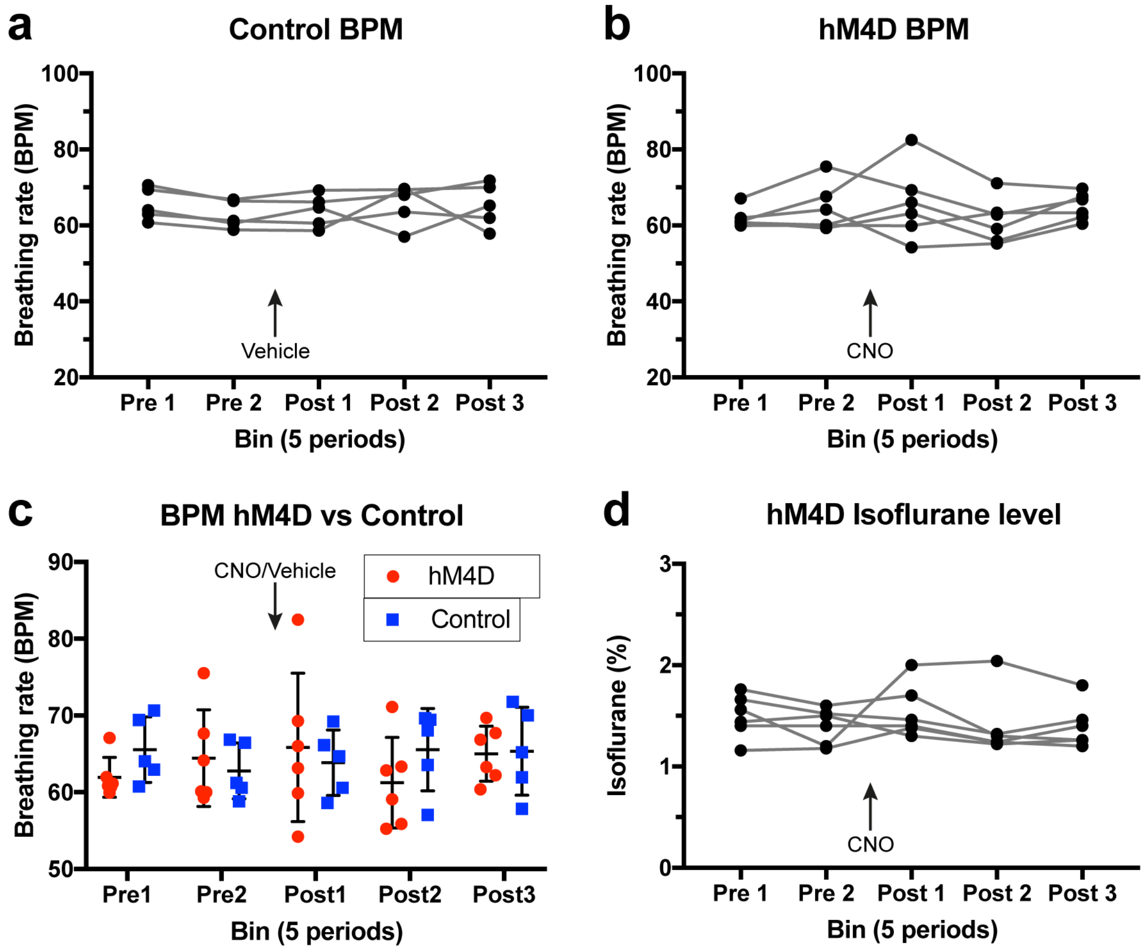

Figure S2. Analysis of breathing rate and Isoflurane level in vehicle-injected and CNO-injected CaMKII-ChR2 mice. **A-B** Breathing rates sampled from 90 s segments preceding each pulse-train, and binned per 5 stimulations, aligned by IP injection of vehicle/CNO. **C** Data from A-B grouped for 2-way ANOVA analysis with Dunnett's multiple comparisons test. There were no significant differences overall (interaction P=0.22, F=1.49) nor for either treatment (P=0.73, F=0.13) or time (P=0.77, F=0.45). **D** Even given maintained anaesthesia depth, isoflurane levels might have been decreased as a compensatory measure if clozapine did in fact still cause increased sedation. However, no such change was seen overall (repeated measures ANOVA, P=0.46, F=0.75) nor comparing the pre-injection bin with the last bin: 1.40±0.17 % vs 1.40±0.22 % (Dunnett's multiple comparisons test, P=0.99). Additionally, comparing normalized post-injection bins of isoflurane level between hM4D and control, no difference was detected: last bins (Post 3), 1.02±0.25 vs 0.88±0.06 (one-way ANOVA, overall P=0.43, with Sidak's multiple comparisons test, P=0.66). Note that one animal was administered increased isoflurane after injection in correspondance with increased breathing rate seen in B (>80 BPM in the Post1 bin).

Figure S3 (of 3)

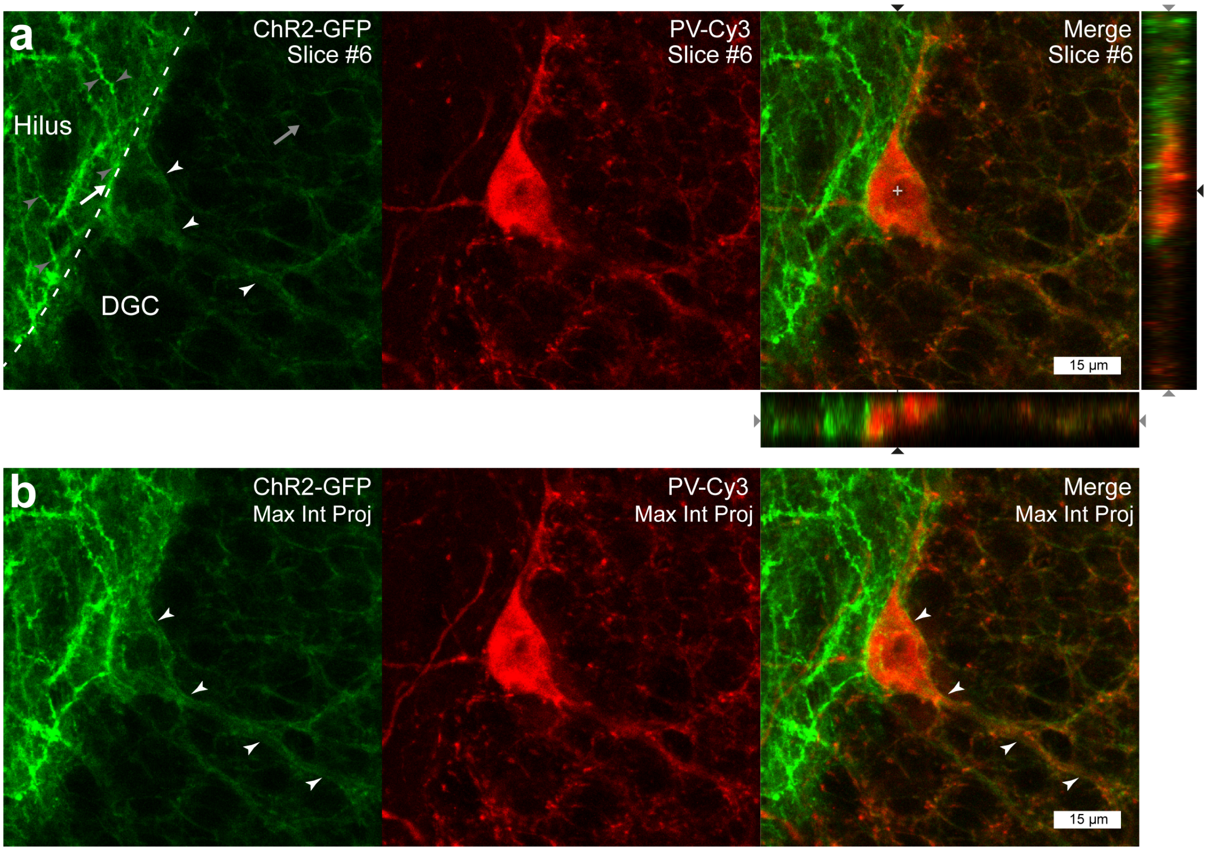

Figure S3. Plausible ChR2 expression in a Thy1-ChR2 mouse PV interneuron. **A** One slice from the confocal z-stack running through the middle of a PV interneuron located at the border between hilus and the DGC layer in the dentate gyrus, highlighting features consistent with PV-neuron expressing ChR2. The right-most image include orthogonal views (XZ, bottom; YZ, right) of the full z-stack (10 slices), intersecting the center of the PV-neuron nucleus, indicated by white cross and black triangles. Level of the z-slice is indicated by grey triangles. GFP fluorescence exceeding baseline levels are visible in the soma and in a large dendrite originating from the PV neuron (white arrowheads). This is in contrast with both the low fluorescence in the cell nucleus, as expected for PV and ChR2 alike, and the lower GFP fluorescence in excitatory granule cells of the DGC layer (one example marked with grey arrow). Granule cells in Thy1-ChR2 mice, despite being known to strongly express ChR2 present in processes e.g. in the molecular layer (compare with images in Fig 3a-c), only weakly contain ChR2 in the cell soma (and then predominately in the cell membrane, outlining the cell). It is unlikely that the uniform GFP fluorescence seen at the membrane of the PV neuron is from afferent excitatory inputs, as nearby excitatory processes (with no trace of PV co-staining) display interspersed strong rounded shapes (examples in the hilus, grey arrowheads), consistent with boutons. Such an excitatory afferent also appears to interface

with the left side of the PV neuron (white arrow). **B** Maximum intensity projection of four z-slices, best representing the extent of the PV neuron soma in A. Putative membrane location of ChR2 in the PV neuron is suggested by the mainly uniform “halo” appearance around and along the soma and dendrite (white arrowheads), which again is in contrast with the interspersed rounded, bouton-like shapes at various locations on the soma and dendrite, more indicative of excitatory inputs.
